# External validation of the parsimonious EuroLung risk models: analysis of the Brazilian Lung Cancer Registry

**DOI:** 10.36416/1806-3756/e20240226

**Published:** 2024-09-16

**Authors:** Paula Duarte D’Ambrosio, Ricardo Mingarini Terra, Alessandro Brunelli, Leticia Leone Lauricella, Carolina Adan Cavadas, Jaqueline Schaparini Fonini, Jefferson Luiz Gross, Federico Enrique Garcia Cipriano, Fabio May da Silva, Paulo Manuel Pêgo-Fernandes

**Affiliations:** 1. Instituto do Câncer do Estado de São Paulo - ICESP - Hospital das Clínicas de São Paulo, Faculdade de Medicina, Universidade de São Paulo, São Paulo (SP) Brasil.; 2. Department of Thoracic Surgery, St. James’s University Hospital, Leeds, United Kingdom.; 3. Centro de Referência Pulmão e Tórax, AC Camargo Cancer Center, São Paulo (SP) Brasil.; 4. Hospital das Clínicas, Faculdade de Medicina de Ribeirão Preto, Ribeirão Preto (SP) Brasil.; 5. Departamento de Cirurgia, Universidade Federal de Santa Catarina, Florianópolis (SC) Brasil.

**Keywords:** Quality of health care, Models, statistical, Public health, Morbidity, Lung neoplasms

## Abstract

**Objective:**

The purpose of this study was to assess performance in the Brazilian Lung Cancer Registry Database by using the parsimonious EuroLung risk models for morbidity and mortality.

**Methods:**

The EuroLung1 and EuroLung2 models were tested and evaluated through calibration (calibration plot, Brier score, and the Hosmer-Lemeshow test) and discrimination (ROC AUCs), in a national multicenter registry of 1,031 patients undergoing anatomic lung resection.

**Results:**

The evaluation of performance in Brazilian health care facilities utilizing risk-adjustment models, specifically EuroLung1 and EuroLung2, revealed substantial miscalibration, as evidenced by calibration plots and Hosmer-Lemeshow tests in both models. In terms of calibration, EuroLung1 exhibited a calibration plot with overlapping points, characterized by a slope of 1.11 and a Brier score of 0.15; the Hosmer-Lemeshow test yielded a statistically significant p-value of 0.015; and the corresponding ROC AUC was 0.678 (95% CI: 0.636-0.721). The EuroLung2 model displayed better calibration, featuring fewer overlapping points in the calibration plot, with a slope of 1.22, with acceptable discrimination, as indicated by a ROC AUC of 0.756 (95% CI: 0.670-0.842). Both models failed to accurately predict morbidity and mortality outcomes in this specific health care context.

**Conclusions:**

Discrepancies between the EuroLung model predictions and outcomes in Brazil underscore the need for model refinement and for a probe into inefficiencies in the Brazilian health care system.

## INTRODUCTION

In a managed care system, the assessment of care quality within surgical units is crucial. Quality is an abstract concept often measured through various indicators.^1^ In thoracic surgery, outcome measures are the main quality indicators. Evaluating the performance of health care providers requires adjusting outcomes for different case mixes across institutions.^2^

To facilitate equitable comparative audits, The European Society of Thoracic Surgeons (ESTS) Database Committee developed risk-adjustment models for morbidity and mortality from a dataset of nearly 50,000 patients.^3^ These models were simplified into the parsimonious EuroLung1 and EuroLung2 versions in 2019.^4^ Those versions offer excellent discrimination capabilities in Europe and are applicable for risk-adjusted performance audits, aiding in quality improvement.

The Brazilian Lung Cancer Registry, a multicenter prospective database, collects data from thoracic procedures at health care facilities in Brazil, supporting quality management. Predictive models like the parsimonious EuroLung risk models facilitate the initial quality assessment and subsequent improvements. Although these models have shown validity in Europe,^4^ they have been shown to have limited discrimination capacity when applied to patients in Canada and Japan.^5^ ^,^ ^6^. To our knowledge, there have been no studies evaluating their applicability in Latin America. This is crucial because of disparities among these populations, including variations in socioeconomic factors and challenges related to diagnosing lung cancer and initiating treatment, often due to barriers to health care access.

The primary objective of this study was to assess the performance of thoracic surgery facilities in Brazil by using the parsimonious EuroLung1 and EuroLung2 risk models within the Brazilian Lung Cancer Registry. A secondary objective was to test the external validity of the parsimonious EuroLung risk models in the Brazilian context.

## METHODS

### Ethics statement

This study was approved by the local institutional review board (Registration no. 16424413.2.1001.0065). The requirement for informed consent was waived because only anonymized data were used.

### Modeling cohort - parsimonious EuroLung1 and EuroLung2 models

In 2017, the ESTS Database Committee published the first models for the prediction of risk after anatomical lung resection (EuroLung1 for cardiopulmonary morbidity and EuroLung2 for 30-day mortality), based on data from approximately 50,000 patients.^3^ A recent update described models that are more parsimonious.^4^ The parsimonious EuroLung models contain five variables for morbidity and six variables for mortality. The two models (EuroLung1 and EuroLung2) contain some common variables associated with morbidity and mortality-age, sex, postoperative FEV_1_ (ppoFEV_1_), and thoracotomy-together with some that are specific for either morbidity (extended resection) or mortality (BMI and pneumonectomy).^4^

Cardiopulmonary complications listed in the ESTS database were included as outcome variables.^7^ Mortality was defined as any death within 30 days after operation or surgical death occurring at any time during the same hospital stay. Extended resection^3^ consisted of chest wall involvement; Pancoast tumors; resection of the atrium, superior vena cava, aorta, diaphragm, or vertebra; bronchial sleeve resection; pleuropneumonectomy; sleeve pneumonectomies; and intrapericardial pneumonectomy.

### Aggregate EuroLung2 model

Similar to what was done in the original EuroLung study,^7^ we tested the aggregate version of the EuroLung2 model to be used as a simple risk stratification tool. using ROC analysis, we found the best cutoff values associated with mortality to be as follows^8^: age > 70 years; ppoFEV_1_ < 70%; and BMI < 18.5 kg/m^2^.^8^ A score of 1 point was assigned to the variables with the smallest odd ratios at logistic regression (age > 70 years and ppoFEV_1_ < 70%) and proportionally weighting the four other variables^4^: 2.5 points for male sex, BMI < 18.5 kg/m^2^, and thoracotomy; and 3 points for pneumonectomy.^4^ Patients were grouped into seven risk classes to evaluate incremental risk of mortality.^4^

### Performance evaluation

This study evaluates the performance in Brazilian health care facilities utilizing the EuroLung1 and EuroLung2 risk-adjustment models.^4^ We used a validation cohort from the nationwide multicenter registry known as the Brazilian Lung Cancer Registry. This registry stands as a forward-looking, comprehensive database including patients who have undergone surgical treatment for lung cancer. It involves 12 institutions across five Brazilian states that have provided data related to patients treated between December of 2009 and December of 2022. Our sample comprised 1,031 lung cancer patients who underwent anatomic lung resection during that timeframe, representing 46.25% of all anatomic lung resections cataloged in the Registry. We excluded patients for whom any values pertaining to pivotal variables were missing.

The definitions of variables were derived from the ESTS standardization document.^9^ The goal is to use both risk models as instruments of internal auditing and for quality control in the local context.

### Statistical analysis

To test the parsimonious EuroLung1 and EuroLung2 scores, we used the published coefficients for both scores^8^ to assess the calibration and discrimination.^9^ ^,^ ^12^ ^,^ ^13^ The logit of the EuroLung1 model was as follows: 

−2.852 + 0.021 ×*age* + 0.472 × *male* − 0.015 ×*ppoFEV1* + 0.662 × *thoracotomy* + 0.324 × *extended resection*

The logit of the EuroLung2 model was as follows: 

−6.350 + 0.047 × *age* + 0.889 ×*male* − 0.055 × *BMI* − 0.010 × *ppoFEV1* + 0.892 × *thoracotomy* + 0.983 × *pneumonectomy*

In our assessment, we employed calibration plots, the Brier score, and the Hosmer-Lemeshow test. The calibration plot displays the relationship between observed frequencies and predicted probabilities.^8^ ^,^ ^10^ ^,^ ^11^ The Brier score quantifies the overall disparity between the predicted probability of an event (such as winning) and the actual occurrence of that event.^8^ ^,^ ^10^ ^,^ ^11^ The Hosmer-Lemeshow test divides the study cohort into deciles based on predicted values, comparing the observed rates with the expected rates.^8^ ^,^ ^10^ ^,^ ^11^ Model discrimination was characterized by the ROC AUC.^8^ ^,^ ^10^ ^,^ ^11^

In order to investigate the linear association between the levels of the score for the variable “risk class” and patient mortality (aggregate EuroLung2 model), the Mantel-Haenszel chi-square test (MH χ^2^) was applied to the data.

Continuous variables are expressed as median and interquartile range, whereas categorical covariates were described as absolute counts and percentages. The 95% confidence intervals are also presented.

Analyses for model development and validation were performed using the R package, version 3.3.3 (R Core Team, 2017) and Stata software, version 15.0 (Stata Corp., College Station, TX, USA). Values of p < 0.05 were considered statistically significant.

## RESULTS

Among 1,210 patients who underwent lung resection and were characterized in our database, critical data were missing for 179, and the remaining 1,031 patients were included in further analyses. The characteristics of the included patients are shown in Table 1. Major cardiopulmonary complications occurred in 196 patients (19.0%), and 46 patients (3.8%) died in the hospital or within the first 30 days after the procedure. The observed morbidity rate was higher than that predicted by the EuroLung1 model (19.0% vs. 13.1%). As for mortality, the observed rate was higher than that predicted by the EuroLung2 model (3.8% vs 1.5%). The observed and predicted outcomes in the validation dataset from the EuroLung1 and EuroLung2 models are shown in Tables 2 and 3, respectively. 

**Table 1:** Characteristics of the Brazilian Lung Cancer Registry and European Society of Thoracic Surgeons databases.^a^

Variable	Database	
	BLCR	ESTS
	(N = 1,031)	(N = 82,383)
Male gender	471 (45.7)	53,780 (65.0)
Age (years)	65.8 (58.5-65.8)	64.6 (57.6-71.2)
BMI (kg/m^2^)	26.1 (23.1-29.4)	25.1 (22.4-28.3)
Chronic artery disease	77 (7.5)	6,725 (8.2)
Cerebrovascular disease	41 (4.0)	2,434 (3.0)
Chronic kidney disease	30 (2.9)	4,579 (5.6)
Complications	196 (19.0)	12,955 (15.7)
Thoracotomy	383 (37.1)	61,252 (74.0)
ppoFEV_1_ (% of predicted)	66.3 (54.5-77.4)	73.0 (59.0-87.0)
Extended resection	55 (5.3)	4,722 (5.7)
Death within 30 days^b^	46 (3.8)	1,851 (2.2)

BLCR: Brazilian Lung Cancer Registry; ESTS: European Society of Thoracic Surgeons; and ppoFEV_1_: postoperative FEV_1_. ^a^Results are expressed as median and IQR for numeric variables and as count and percentage of the total for categorical variables. ^b^Counted from the date of anatomic lung resection.

**Table 2:** Observed and predicted outcomes from the parsimonious EuroLung1 model in the validation cohort (N = 1,031).

Decile	Probability	Events		No events	
		Observed	Predicted	Observed	Predicted
		(n)	(n)	(n)	(n)
1st	5.84	6	4.7	97	98.30
2nd	7.29	13	6.7	90	96.30
3rd	8.54	12	8.1	91	94.90
4th	9.8	11	9.4	92	93.60
5th	11.47	15	10.8	88	92.20
6th	13.33	22	12.8	81	90.20
7th	15.35	21	14.7	82	88.30
8th	18.24	18	17.3	85	85.70
9th	23.64	33	21.3	70	81.70
10th	40.06	45	29.4	59	74.60

**Table 3:** Observed and predicted outcomes from the parsimonious EuroLung2 model in the validation cohort (N = 1,029).

Decile	Probability	Events		No events	
		Observed	Predicted	Observed	Predicted
		(n)	(n)	(n)	(n)
1st	0.28	0	0.2	103	102.80
2nd	0.4	1	0.4	100	102.60
3rd	0.52	0	0.5	101	102.50
4th	0.69	1	0.6	98	102.40
5th	0.93	3	0.8	93	101.20
6th	1.18	1	1.1	99	101.90
7th	1.6	4	1.4	98	101.60
8th	2.21	3	1.9	97	101.10
9th	3.44	8	2.8	91	100.20
10th	14.06	16	5.7	76	97.30

**Figure 1:**
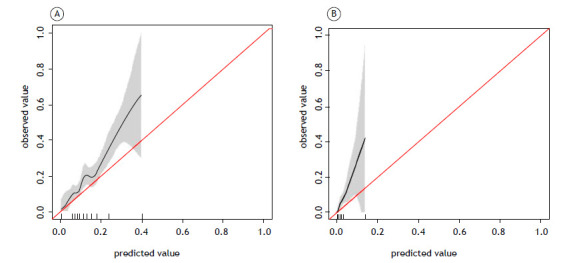
Calibration plot for the EuroLung1 and EuroLung2 models (A and B, respectively). The calibration curves highlighted in gray represent the 95% CIs. EuroLung1 model applied to data from 1,031 patients; EuroLung2 model applied to data from 1,029 patients.

**Figure 2:**
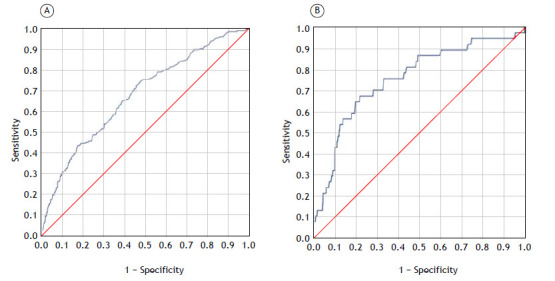
Discrimination. ROC curves for the EuroLung1 and EuroLung2 model analyses (A and B, respectively). AUC for the EuroLung1 model analysis = 0.678 (95% CI: 0.636-0.721). AUC for the EuroLung2 model analysis = 0.756 (95% CI: 0.670-0.842). EuroLung1 model applied to data from 1,031 patients; EuroLung2 model applied to data from 1,029 patients.

For the EuroLung1 model, the calibration plot shows some overlap, indicating a lack of perfect calibration. The slope of 1.11 suggests that the model is slightly overestimating probabilities (Figure 1). The Brier score of 0.15 indicates moderate calibration performance, and the p-value of 0.015 from the Hosmer-Lemeshow test suggests that the model is not well calibrated. In addition, the AUC for the EuroLung1 model was 0.678 (95% CI: 0.636-0.721), indicating weak discrimination performance (Figure 2).

For the EuroLung2 model, the calibration plot shows less overlap, indicating better calibration (i.e., improved alignment between predicted probabilities and observed outcomes) than that of the EuroLung1 model. The slope of 1.22 further supports that finding, suggesting a closer fit between predicted and observed probabilities (Figure 2). The Brier score of 0.03 indicates good calibration performance, although the Hosmer-Lemeshow test suggested that the model is not well calibrated, given the p-value of 0.044. The EuroLung2 model had acceptable discrimination, as demonstrated by an AUC of 0.756 (95% CI: 0.670-0.842).

Patients were grouped into five risk classes showing incremental risk of mortality, as can be seen in Table 4. There is a statistically significant linear association (p < 0.001; MH χ^2^ = 6.530, therefore, p < 0.05) between the levels of the score of the aggregate EuroLung2 model and the percentage of mortality of patients. The patients in the lowest risk class had a 3.4% mortality rate, whereas those in the highest risk class had a 28.2% mortality rate. It is noteworthy that the 9.5-12.0 score category was removed from this analysis because it comprised only five cases, which is not sufficient for a reliable prognosis of death.

**Table 4:** Analysis of linear association between the variables risk class and mortality from the aggregate EuroLung2 model in the sample as a whole (N = 1,205).

Risk class (score category)	Patients	Deaths	Mortality rate	95% CI
	(n)	(n)	(%)	
0-2.5	589	20	3.40	(3.13-3.66)
3.0-5.0	407	26	6.39	(5.81-6.97)
5.5-6.5	123	18	14.63	(12.43-16.84)
7.0-7.5	47	9	19.15	(21.85-34.56)
8.0-9.0	39	11	28.21	(5.98-34.02)

Note: p < 0.001 (Mantel-Haenszel chi-square = 6.530) between risk class and mortality.

## DISCUSSION

The external validation assessment of the parsimonious EuroLung1 and EuroLung2 models reveals miscalibration in both. In addition, performance assessments of Brazilian health care facilities using risk-adjustment models like EuroLung1 and EuroLung2 indicate a higher observed mortality and morbidity rate in the Brazilian Lung Cancer Registry than those predicted by the EuroLung risk models. The miscalibration observed in both models indicates the limitations of directly applying them to the Brazilian population without appropriate adjustments, and it emphasizes the need for recalibration or development of locally tailored models to enhance accuracy and improve clinical decision-making. These findings also suggest limitations in the direct application of the EuroLung models to the Brazilian population without suitable modifications, which could potentially highlight the underperformance of health care facilities in Brazil.

The EuroLung risk models represent recent advancements in population-based tools for predicting cardiopulmonary morbidity and mortality following anatomic lung resection, necessitating external validation across diverse populations for generalizability.^2^ ^,^ ^3^ However, such validation is often hindered by population-specific discrepancies.^8^ In the Brazilian cohort, the EuroLung2 model demonstrated acceptable discrimination, as evidenced by a higher AUC value. However, discrepancies in both models probably stem from the exclusion of critical variables in the ESTS model, which are vital in the Brazilian context, such as racial and social factors, along with caseload variations. This observation is consistent with the findings of a study conducted in Japan,^6^ highlighting the predictive limitations of the EuroLung models for morbidity and mortality due to notable baseline differences with the European demographic.^6^ Such omissions might significantly impact the observed underperformance of Brazilian health care facilities. Nonetheless, the discrepancy between the observed and predicted morbidity rates can be attributed to patient-specific factors, which encompass pre-existing comorbidities, socioeconomic conditions, and the disease stage at the time of diagnosis.^9^ ^,^ ^12^ ^-^ ^14^ In Brazil, a middle-income country, the absence of adequate education regarding disease prevention often results in patients presenting to the health care system with advanced, symptomatic disease,^15^ in contrast to their counterparts in high-income countries. Notably, Knorst et al.^16^ reported a historical cohort study in which the time from the onset of initial symptoms to the diagnosis of lung cancer in a university hospital in the southern region of Brazil exceeded 20 weeks, whereas the Standing Medical Advisory Committee recommendation is that the interval between symptom onset and treatment should be no longer than 6-8 weeks.

The discrepancy in mortality may be linked to systemic factors, including access to health care services for prevention, timely diagnosis, and treatment.^17^ In Brazil, over 75% of patients depend exclusively on the Brazilian Unified Health Care System. Despite its goal of providing universal care, the system faces significant challenges related to accessibility, diagnostic delays, treatment availability, and substantial disparities among cancer care facilities concerning diagnostic and treatment technologies.^18^ ^,^ ^19^ For example, Lista et al.^20^ discovered that almost 80% of the initial treatments for lung cancer in Brazil did not take the diagnosis into consideration; only 6.8% of patients received a lung cancer diagnosis within 30 days after experiencing symptoms. Another study conducted among the Brazilian population revealed that 10-18% of lung cancer patients, regardless of their disease stage, did not undergo any cancer treatment due to their poor clinical condition,^21^ rendering them unable to withstand the risks associated with treatment.

Lung cancer remains a pressing public health concern in Brazil, and as a response to this challenge, the country has implemented a series of public policies aimed at improving surgical treatment outcomes. Over the past decade, Brazil has made significant strides in this area, with initiatives focused on expanding access to early detection, enhancing surgical techniques, and ensuring equitable care for all patients. In addition, strong public health measures in Brazil have led to notable reductions in tobacco consumption in Brazil, setting a valuable precedent for other low- and middle-income countries. National research in Brazil has revealed a nearly 50% reduction in smoking prevalence, aligning with a corresponding decrease in tobacco-related fatalities.^22^ These policies, coupled with efforts to reduce health care disparities, have the potential to revolutionize lung cancer surgery in Brazil, ultimately leading to better patient outcomes and a brighter future in the fight against this devastating disease.

Another reason for the underperformance of Brazilian health care facilities may be related to surgical skills. Therefore, we will examine the data in a more granular manner to gain a deeper understanding of the quality of surgical care at the facilities that could be associated with these outcomes. Subsequently, we will investigate design actions aimed at enhancing improvement factors. Overall, these findings highlight the complex interplay between patient-specific and systemic factors that influence the calibration and performance of risk models in a diverse health care landscape such as that of Brazil. Further research and tailored interventions are essential to bridge these disparities and improve the quality of lung cancer care in the country.

The present study relied on data from the Brazilian Lung Cancer Registry, a prospective multicenter database. The main limitation of the study is the size of the sample, which was small in comparison with the original population from which the models were generated. In addition, the study may simply be underpowered to assess the calibration and discrimination of the risk models. The fact that 46% of the cases were excluded from analysis in both arms because key values were missing raises concerns about the validity of our findings. This significant data gap suggests a potential bias, given that less than half of the facilities contributed meaningful data, limiting the comprehensiveness and reliability of the analysis. Furthermore, the Brazilian Lung Cancer Registry includes 12 institutions in five Brazilian states and does not represent the entire country. However, it is important to note that it stands as the only database related to the surgical treatment of lung cancer in Brazil. Therefore, the findings should be interpreted within the context of the studied population. Moreover, our database initially included mostly patients from the public health care sector, only later including those from the private sector. In the present study, no analyses were carried out separating patients by sector. 

The disparities between the EuroLung model predictions and Brazilian patient outcomes highlight the need for model adjustments and signal potential underperformance within the health care system in Brazil, underscoring the importance of investigating contributing factors. The EuroLung2 model showed promising performance in terms of discrimination in the Brazilian cohort, indicating its potential utility. Considering additional variables and exploring machine learning analytics may further enhance the performance of surgical risk prediction models.
